# Opposing roles by *KRAS* and *BRAF* mutation on immune cell infiltration in colorectal cancer – possible implications for immunotherapy

**DOI:** 10.1038/s41416-023-02483-9

**Published:** 2023-12-01

**Authors:** Sofia Edin, Björn Gylling, Xingru Li, Åsa Stenberg, Anna Löfgren-Burström, Carl Zingmark, Bethany van Guelpen, Ingrid Ljuslinder, Agnes Ling, Richard Palmqvist

**Affiliations:** 1 Department of Medical Biosciences, Pathology, Umeå University, Umeå, Sweden; 2 Department of Radiation Sciences, Oncology, Umeå University, Umeå, Sweden; 3 Wallenberg Centre for Molecular Medicine, Umeå University, Umeå, Sweden

**Keywords:** Colorectal cancer, Cancer immunotherapy

## Abstract

**Background:**

The immune response has important clinical value in colorectal cancer (CRC) in both prognosis and response to immunotherapy. This study aims to explore tumour immune cell infiltration in relation to clinically well-established molecular markers of CRC.

**Methods:**

Multiplex immunohistochemistry and multispectral imaging was used to evaluate tumour infiltration of cytotoxic T cells (CD8^+^), Th1 cells (T-bet^+^), T regulatory cells (FoxP3^+^), B cells (CD20^+^), and macrophages (CD68^+^) in a cohort of 257 CRC patients.

**Results:**

We found the expected association between higher immune-cell infiltration and microsatellite instability. Also, whereas *BRAF*-mutated tumours displayed increased immune-cell infiltration compared to *BRAF* wild-type tumours, the opposite was seen for *KRAS*-mutated tumours, differences that were most prominent for cytotoxic T cells and Th1 cells. The opposing relationships of *BRAF* and *KRAS* mutations with tumour infiltration of cytotoxic T cells was validated in an independent cohort of 608 CRC patients. A positive prognostic importance of cytotoxic T cells was found in wild-type as well as *KRAS* and *BRAF*-mutated CRCs in both cohorts.

**Conclusion:**

A combined evaluation of MSI status, *KRAS* and *BRAF* mutational status, and immune infiltration (cytotoxic T cells) may provide important insights to prognosis and response to immunotherapy in CRC.

## Background

Colorectal cancer (CRC) is one of the major malignancies in the world and the second-leading cause of cancer deaths [1], medical advances notwithstanding. The basis for curative therapy is surgical resection, but still around 40% of the patients die from metastatic disease. In addition to the TNM staging system, the tumour immune response has been found an important prognosticator in CRC, with a higher density of tumour-infiltrating lymphocytes predicting a better outcome [2]. This finding has led to a joint effort to introduce the Immunoscore, based on immunohistochemical (IHC) evaluation of T cell markers, into clinical practice [3]. T cells are known combatants in anti-tumour immunity and can inhibit tumour growth by direct killing (cytotoxic T cells) [4]. The activity of the cytotoxic T cells is, however, tightly regulated by the balance of other T cell subsets; the T helper 1 (Th1) and T helper 2 (Th2) cells, and the T regulatory cells, as well as the expression of checkpoint inhibitors, e.g., CTLA4 and PD1/PDCD1 [5].

Considering the well-established prognostic value of the tumour immune response in CRC, these patients should be promising candidates for immunotherapy. However, the clinical response to immune checkpoint inhibitors has so far been quite limited in patients with metastatic CRC, the strongest effect found in patients with microsatellite instable (MSI) tumours [6]. The Food and Drug Administration has recently approved Pembrolizumab and Nivolumab (PD1 inhibitors) for clinical use in the treatment of metastatic MSI CRC patients, but ongoing studies suggest a striking beneficial effect of immune checkpoint inhibitor therapy also in non-metastatic MSI CRC [7, 8].

Sporadic CRC is a heterogenous disease, and the heterogeneity is partly seen in its development through three major genetic pathways: The classical chromosomal instability adenoma to carcinoma pathway responsible for approximately 70% of sporadic CRCs, the MSI pathway caused by aberrations in mismatch repair genes found in around 15% of CRCs, and the epigenetic instability CpG island methylator phenotype pathway causing 30–40% of sporadic CRCs. These pathways are partly driven by mutations in tumour oncogenes and are not mutually exclusive. Some of the most common driver mutations found in sporadic CRC are activating mutations in *KRAS* accounting for 30–40% of all CRCs and activating mutations in *BRAF* which occur in 5–15 % of the tumours [9].

Many robust studies have linked immune cell infiltration to an immunogenic MSI/d-MMR tumour status [4, 10]. However, the Immunoscore has been shown to be a stronger predictor of patient survival than tumour MSI status, suggesting that there are other subgroups of CRC patients in whom immune cell infiltration has clinical value [11]. Indeed, studies by us and others have proposed that there are microsatellite stable (MSS) tumours with a similar tumour immune profile as MSI tumours, e.g., the *POLE* mutated CRCs [11–14]. In addition, the expression of immune checkpoint proteins such as PD1 and PD-L1/CD274 are not restricted to MSI tumours [15, 16]. More comprehensive studies on the distribution of immune cell subsets according to *KRAS* and *BRAF* mutation are rare. *KRAS* and *BRAF* mutations are mainly mutually exclusive [17]. *BRAF* mutation has been associated with MSI tumours, but is also found in MSS tumours [18]. *BRAF-*mutated tumours have also been linked to a higher immune cell infiltration and expression of immunotherapeutic targets [19], but the relative contribution of mutated *BRAF* and MSI to the immune response remains uncertain. The potential of extracting putative predictive information from *KRAS* and *BRAF* mutation analyses in immunotherapy would be attractive since these are often already included, in addition to MSI status, in the clinical assessment of CRC patients.

This project aims to better characterise the local immune response to CRC in association with molecular characteristics. In particular, the importance of *KRAS* and *BRAF* mutation on immune cell infiltration will be evaluated. An increased understanding of the distribution of the immune response in different molecular subgroups of CRC may lead to the identification of prognostic and predictive tools, as well as new targets for therapy.

## Materials and methods

### Study cohort of patients with CRC

A logistic system has been developed for collecting biological samples and data from CRC patients diagnosed at Umeå University Hospital, Umeå, Sweden. Since 2010, CRC patients are enroled in U-CAN (Uppsala-Umeå Comprehensive Cancer Consortium) [20], which longitudinally collects blood, tissue, faeces, radiological data, and clinical data from all enroled patients. The present study utilised retrospectively collected resected formalin-fixed paraffin-embedded (FFPE) primary tumour tissue specimens from a cohort of stage I–IV U-CAN patients with faecal samples collected between the years 2010–2014 (*n* = 257), for studies of microbial markers in relation to molecular subtypes, immune cell infiltration and prognosis [21]. The patient tumour tissue specimens of this cohort have been analysed for molecular changes including *BRAF*^*V600E*^ mutation and *KRAS* mutations (codon 12 and 13), and MSI status, as previously described [22]. Also data on clinicopathological characteristics, and survival have been collected. From this cohort a tumour tissue microarray (TMA) was prepared in September 2020, from patients operated on in Umeå with available tumour tissue at that time (*n* = 151). The TMA blocks were constructed using a TMA GRAND Master instrument (3DHISTEC, Budapest, Hungary) by punching 1 mm cores from archived FFPE tissue samples. The TMAs used for this study, included two cores of tumour tissue taken at the tumour front and one core taken from the tumour centre for each patient.

For validation, retrospectively collected tumour specimens from CRC patients identified within a population-based cohort from northern Sweden, the Northern Sweden Health and Disease Study (NSHDS), were used. The NSHDS cohort has been described in detail previously [23]. Patients diagnosed between the years 2000–2016 with available tumour tissue for analyses of molecular traits and immune infiltration were included in this study (*n* = 608). Clinical patient characteristics can be found in Supplementary Table S1.

### Multispectral imaging for in situ evaluations of immune cell subsets in CRC

We evaluated immune cell infiltration using the Vectra® system for multispectral quantitative automated pathology imaging (Akoya Biosciences). The multiplexed IHC staining was a modification of the Opal^TM^ 7 Solid Tumour Immunology Kit (Akoya Biosciences). Tissue TMA slides were sequentially stained using antibodies against T-bet, CD8, CD20, FoxP3, CD68 and pan-Cytokeratin. The CD4 antibody used in the kit was exchanged by T-bet (clone H210, Santa Cruz Biotechnology Inc.). The CD8 antibody in the kit was exchanged by CD8 (clone 144b, DAKO). The final concentrations of antibodies and Opal dyes used can be found in Supplementary Table S2. The Prolong Diamond Antifade Mountant (ThermoFisher, Waltham, MA, USA) was used to mount slides. Multispectral imaging was performed using the VECTRA 3 Quantitative Pathology Imaging System (Akoya Biosciences) as previously described [24]. A spectral library was created and applied to the images for spectral unmixing together with an autofluorescence control using the inForm^®^ software (Akoya Biosciences, Marlborough, USA). The signal intensity of each marker was adjusted to give a signal range of 5–30 ms at exposure times of 30–200 ms. For validation, multiplex images were compared to monoplex images and inspected for interference and cross-talk. The inForm^®^ software (Akoya Biosciences) was further used to quantify the different immune cells in tissue segmented areas using machine learning algorithms, as previously described [24]. T-bet was used as a marker for T helper 1 cells, but has been reported to be expressed also in cytotoxic (CD8 positive) T cells [25]. In this study, CD8 positive cells generally showed no or low expression of T-bet and were classified as cytotoxic T cells. Remaining T-bet positive cells were defined as T helper 1 cells. Tissue was segmented into tumour epithelial area, stromal area, tumour debris and no tissue. Extracellular mucin was classified as tumour debris. Exclusions were, large part of or whole core lost, lack of tumour or stromal tissue, bad quality of staining, and heavy necrosis. Image analysis was supervised by two experienced pathologists, and the investigators were blinded from patient data during the evaluations. Cell segmentation summary data was collected for each TMA core as number of individual cell types per mm^2^ (tumour or stromal compartment). For patients with data on two TMA cores, data was given as an average number of cells per mm^2^. Data was successfully collected from tumour front of 139 patients (of which 33 had data from only one core), and from tumour centre of 137 patients. Infiltrating immune cells, as identified by the respective markers, were further divided into groups of high and low infiltration with the median number of infiltrating cells for all CRC cases used as cut-off. Patients not surgically resected for CRC and patients dying from post-operative complications within 90 days of surgery were excluded from survival analyses.

### Evaluation of cytotoxic T cells by immunohistochemistry for validation

The evaluation of cytotoxic T cells (CD8^+^) by immunohistochemistry in the NSHDS validation cohort has been previously described [23]. In brief, CD8^+^ cells were stained on whole tissue sections and semi-quantitatively evaluated at the tumour front of CRC specimens (*n* = 608) ranging from score (1) no or sporadic, (2) moderate numbers, (3) abundant or (4) highly abundant. Patients with pre-operatively irradiated rectal tumours were excluded (*n* = 98), leaving in total 510 CRC tumour specimens for analyses.

### Statistics

IBM SPSS Statistics v.28 (SPSS Inc.) was used for statistical analyses. The distribution of each variable and subgroup was assessed and appropriate statistical tests chosen accordingly. Comparisons of differences within continuous variables between groups were hence performed by non-parametric two-sided Mann–Whitney *U* tests or Kruskall-Wallis *H* tests. The χ^2^ test or Fischer´s exact test (when expected counts were less than 5) was used for comparisons of categorical variables. Cancer-specific survival was defined as death with disseminated or recurrent disease and estimated using Kaplan–Meier survival analyses. Follow-up time was from day of surgery until death or end of follow-up (October 2021 for U-CAN, May 2022 for NSHDS). Median follow-up time was 92.8 months for U-CAN and 94.5 months for NSHDS. Log-rank tests were used to estimate differences in 5-year cancer-specific survival between groups. The Cox Proportional-Hazards model was used for uni- and multivariable survival analyses. A *P* value < 0.05 was considered statistically significant.

## Results

### Distribution of different immune cells in tumour front and centre of CRC patients

**Fig. 1:**
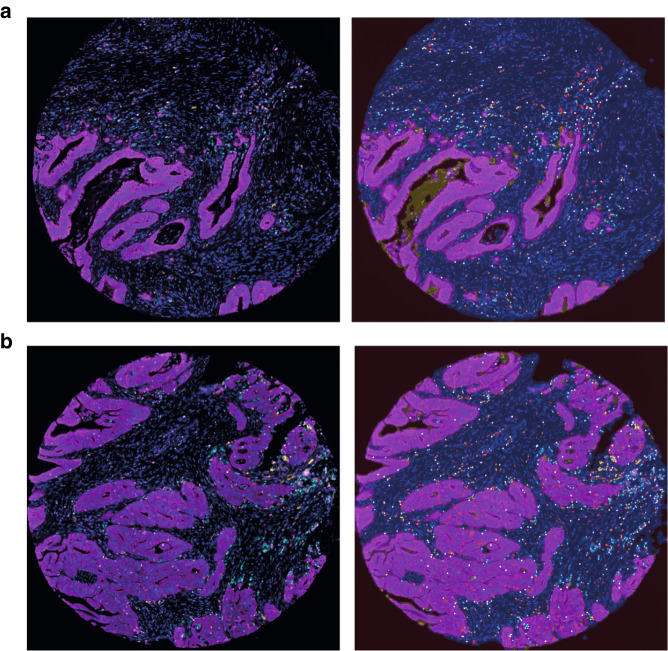
Multispectral imaging of immune cell infiltration in tumour front and centre of CRC study patients. Shown are high-magnification areas (20x) of representative multiplex IHC stains from (**a**) a TMA (1 mm core) taken at the tumour front and (**b**) a TMA (1 mm core) taken at the tumour centre. Left panel displays multiplex IHC images after spectral unmixing, of cytokeratin (magenta), CD8^+^ cytoxic T cells (red), T-bet^+^ Th1 cells (white), FoxP3^+^ T regulatory cells (orange), CD20^+^ B cells (yellow), and CD68^+^ macrophages (cyan). Right panel displays the same multiplex IHC but with tumour tissue segmentation map of stromal area (blue), tumour epithelial area (magenta), tumour debris (yellow) and no tissue (brown), and the cell phenotypes from which data was collected highlighted using the above described colours.

We have set up a functional assay for multiplex immunohistochemistry and evaluation of CD8^+^ cytotoxic T cells, T-bet^+^ Th1 cells, FoxP3^+^ T regulatory cells, CD20^+^ B cells, CD68^+^ macrophages, and cytokeratin using the VECTRA system for multispectral imaging (Fig. 1). We have evaluated this immune cell infiltration panel in surgically resected stage I–IV patients from a CRC patient study cohort (*n* = 257). The different immune cells were evaluated in the stromal compartment and in the tumour compartment at the tumour front and in the tumour centre. The infiltrating immune cells were shown to be quite evenly distributed between the tumour front and the tumour centre, but with higher levels in the stromal area compared to the tumour epithelial area (Table 1). Data from the tumour epithelial area for FoxP3^+^ T regulatory cells and CD20^+^ B cells was not further analysed since these immune cells were rarely present within this compartment.

**Table 1:** Distribution of immune cells at the tumour front and centre in CRC tumours.

Shown are median numbers of infiltrating cells/mm^2^ stromal tissue or tumour epithelial tissue within the tumour front or centre.

### Analyses of immune cell infiltration according to patient clinical and pathological characteristics

The strongest associations of immune cells to clinical and pathological parameters in study patients were found at the tumour front (Table 2). A slightly higher infiltration of immune cells was found in women, especially within the tumour epithelial area. Immune cell infiltration was also strongly linked to the right-sided colon cancers, except for B cells and macrophages. All immune cell types were found linked to tumour stage, with a gradual decrease in immune cell infiltration from stage I to stage IV tumours (Table 2). Pre-operative radiotherapy of rectal cancers was strongly associated with a decreased infiltration of immune cells. Thus, for further analyses, the pre-operatively irradiated rectal cancers were excluded. The clinical and pathological associations of immune cell infiltration in the tumour centre can be found in Supplementary Table S3.

**Table 2:** Associations of immune cells in tumour front to clinicopathological characteristics of CRC patients.

Shown are median numbers of infiltrating cells/mm^2^ stromal tissue or tumour epithelial tissue within the tumour front.

*RT* radiotherapy.

*Indicates significant *P* values (*P* < 0.05) according to Mann–Whitney *U* or Kruskall-Wallis *H* tests.

### Analyses of immune cell infiltration in molecular subgroups of CRC

Infiltrating immune cells at the tumour front of study patients further exhibited several significant associations with tumour molecular subtypes (Table 3). The MSI tumours were found to be significantly more infiltrated by cytotoxic T cells, Th1 cells, and macrophages, in validation of our analyses. We found contrasting associations of immune cell infiltration in *KRAS-*mutated and *BRAF-*mutated CRC tumours. While *KRAS-*mutated tumours showed less infiltration of immune cells compared to wild-type *KRAS* tumours, in particular cytotoxic T cells and Th1 cells, *BRAF-*mutated tumours showed a higher infiltration of these immune cells compared to wild-type *BRAF* tumours. No associations to *KRAS* or *BRAF* mutation was found for T regulatory cells at the tumour front. The associations of immune cells in the tumour centre to molecular characteristics can be found in Supplementary Table S4. Similar, but weaker associations were found in the tumour centre. However, in the tumour centre *KRAS* mutation was significantly associated with increased infiltration of regulatory T cells (Supplementary Table S4).

**Table 3:** Associations of immune cells in tumour front to molecular characteristics of CRC tumours.

Pre-operatively irradiated rectal cancers were excluded. Shown are median numbers of infiltrating cells/mm^2^ stromal tissue or tumour epithelial tissue within the tumour front.

*Indicates significant P values (*P* < 0.05) according to Mann–Whitney *U* or Kruskall-Wallis *H* tests.

To investigate the relative contribution of *BRAF* mutation and MSI status on immune cell infiltration, we stratified the analysis of immune cell infiltration according to *BRAF* mutation by MSI status (Table 3). Even though limited by sample size, *BRAF* mutation was found to increase infiltration of cytotoxic T cells in both MSI and MSS tumours to similar levels. In contrast, Th1 cell infiltration appeared to be more linked to MSI status than *BRAF* mutation. However, *BRAF* mutation was still linked to increased infiltration of most analysed immune cells (with the exception of regulatory T cells) in the MSS tumours.

In validation analyses, tumour infiltration of cytotoxic T cells was evaluated in an independent cohort of CRC patients, the NSHDS (*n* = 608). Also here, a contrasting role on immune cell infiltration by *KRAS* and *BRAF* mutation was found. The proportion of *KRAS* mutated tumours was found to decrease with increasing score of infiltrating cytotoxic T cells, while the proportion of *BRAF* mutated tumours instead was found to increase (Table 4). The proportion of MSI tumours was also found to increase with increasing immune score (Table 4). However, even though MSI status was found highly associated with a high immune score, around half of the tumours with the highest immune score were defined as MSS (Table 4). In this cohort where infiltration of cytotoxic T cells was assessed on a semi-quantitative scale (score 1-4), immune cell infiltration by *BRAF* mutation was found mainly linked to MSI tumours (Table 4).

**Table 4:** Associations of cytotoxic T cells in tumour front to molecular characteristics of CRC tumours in the validation cohort.

Shown is the percentage of different molecular subtypes within tumours of immune scores 1-4, where score 1 - no/sporadic; score 2 - moderate; score 3 - abundant; or score 4 - highly abundant. The sum of each box is 100%.

Pre-opereatively irradiated rectal cancers were excluded. Χ^2^ tests were used to calculate *P* values. *P value for linearity.

### Analyses of immune cell infiltration in CRC prognosis

**Fig. 2:**
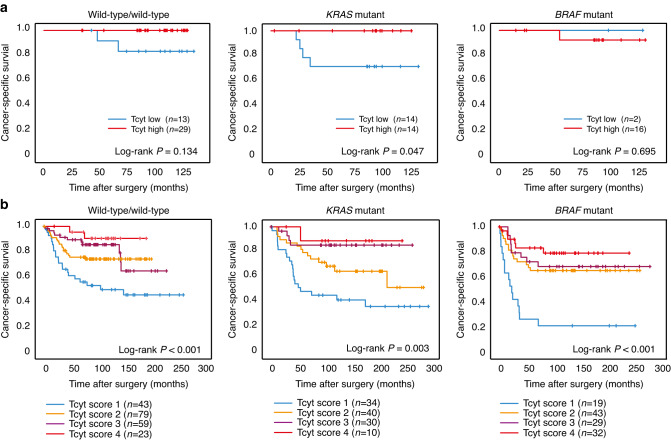
The prognostic importance of cytotoxic T cells at the tumour front in CRC in relation to *KRAS/BRAF* mutation status. Shown are Kaplan–Meier plots of cancer-specific survival in patients with different levels of stromal infiltration of cytotoxic T cells (Tcyt) in wild-type/wild-type tumours, *KRAS* mutant tumours, and *BRAF* mutant tumours, as indicated, in (**a**) the study cohort, and in (**b**) the NSHDS validation cohort. Pre-operatively irradiated rectal cancers were excluded. Log-rank tests were used to calculate differences in 5-year survival between groups. *P* < 0.05 was considered statistically significant.

A positive prognostic effect of immune cell infiltration in CRC tumours has been established in the literature. In this study, even though on a relatively small patient cohort, we also found a significant positive prognostic role for stromal infiltration of cytotoxic T cells, Th1 cells and T regulatory cells at the tumour front of stage I–IV CRC patients, with the strongest association found for cytotoxic T cells (*P* = 0.009) (Supplementary Fig. S1). The prognostic significance remained in stage I-III CRC patients for cytotoxic T cells and T regulatory cells (*P* = 0.047 and *P* = 0.014, respectively). Stratifying the prognostic role of stromal infiltration of cytotoxic T cells by *KRAS* or *BRAF* mutation status in both the study cohort and the NSHDS validation cohort, revealed a prognostic role of cytotoxic T cells independent of *KRAS* and *BRAF* mutation in CRC (Fig. 2). The independent prognostic role of cytotoxic T cells was further tested in a multivariable Cox Proportional-Hazards model in stage I-III patients from the NSHDS cohort. The positive prognostic role of cytotoxic T cells remained statistically significant in a multivariable analysis including gender, age, localisation, tumour stage, *BRAF* mutation, *KRAS* mutation, and MSI status (Supplementary Table S5).

## Discussion

The immune response has important clinical value in both prognosis and therapy of CRC patients. Immune checkpoint inhibitors have proven very effective in some patients with metastatic immunogenic MSI tumours, but there are likely additional CRC patient groups where immunotherapy may have clinical value. CRC is a heterogeneous disease, which calls for careful investigations of the immune response in molecular subgroups of CRC to identify potential predictive markers of response to immunotherapy. Here, we investigated the infiltration of different immune cell subsets in relation to molecular characteristics of CRC, with a focus on the clinically well-established molecular markers *KRAS* and *BRAF* mutation.

We analysed the tumour infiltration of five different immune cells (cytotoxic T cells, Th1 cells, regulatory T cells, B cells and macrophages), both in the tumour centre and at the tumour front of patients with CRC. Immune cells were quite evenly distributed between the tumour centre and tumour front, however the associations to clinicopathological and tumour molecular traits were found to be stronger at the tumour front. We further investigated both the stromal area and the tumour epithelial area, noticing a higher density of immune cells in the stromal compartment, a finding in line with a recent study by Mezheyeuski et al., where they used a similar approach to characterise the immune landscape in CRC [26]. These results support the analysis of immune cell infiltration in TMAs taken at the tumour front as a suitable approach in CRC.

Right-sided colon cancers were more highly infiltrated by different subsets of T cells at the tumour front, a higher immune cell infiltration being a recognised feature of these tumours [27]. We further found a decreasing infiltration of all immune cells analysed from stage I to stage IV tumours, findings that may be related to tumour immune escape and metastasis. Pre-operatively radiated rectal tumours displayed a significantly lower infiltration of all immune cells analysed, which is in line with the reported radiosensitivity of lymphocytic cells [28]. Even though being an interesting patient group, they should likely be analysed separately as pre-operative radiotherapy may alter the anti-tumour immune response, introducing e.g., damage associated molecular patterns and rediscovery of tumour antigens [29]. Pre-operative radiotherapy has so far shown contradictory results in CRC, with both enhanced and reduced immune cell infiltration, calling for further investigation [30–32].

When analysing immune cell infiltration in association with known molecular parameters of CRC tumours, we found the expected and well-established associations of immune cells to MSI tumours [4, 10]. Immunogenomic studies have, however, shown that not only hypermutated tumours (MSI or *POLE* mutated) but also some non-hypermutated MSS tumours display high neoantigen load and tumour immune cell infiltration [33–35]. Interestingly, we here found an opposing role of *KRAS* and *BRAF* mutation on the immune response in CRC. While *KRAS-*mutated tumours displayed decreased immune cell infiltration compared to *KRAS* wild-type tumours, the opposite was found for *BRAF-*mutated tumours, differences that were most obvious for the cytotoxic T cells and the Th1 cells. For cytotoxic T cells, the findings were validated in the NSHDS cohort. A contrasting role of *KRAS* and *BRAF* mutation on immune cell infiltration in CRC tumours has been previously suggested [36, 37]. Ling et al. found a similar contrasting infiltration of Th1 cells according to *KRAS* and *BRAF* mutation in CRC [37], and Lal et al. found that *KRAS-* and *BRAF-*mutated CRCs were found in clusters with low and high expression of immune response genes, respectively [36]. *KRAS-*mutated CRCs have in previous studies been associated with supressed immune pathways and decreased immune cell infiltration [38–40]. *BRAF* mutation has been linked to the immunogenic MSI tumours [27], but our findings partly suggest that *BRAF* and MSI may to some extent have individual effects on the immune response in CRC. Further larger studies are needed to establish the role of *BRAF* mutation on immune cell infiltration in CRC. In a study by Cen et al., where they compared the immune microenvironment in *BRAF-*mutated tumours to that of *BRAF* wild-type tumours, an increased immune cell infiltration was found along with an increased expression of immune checkpoints, e.g., PD1, PD-L1 and CTLA4 [19]. Bolzacchini et al. further identified a gene expression based “hot/inflamed” immunoprofile in a high fraction (52%) of patients with advanced *BRAF*-mutant CRC, with only a partial overlap with MSI [41]. Additionally, 42% of the MSI tumours showed a “cold” immunoprofile. Barras et al. also suggested different subtypes of *BRAF-*mutant CRCs based on gene expression, finding differences in immune regulating genes as well as patient survival, regardless of MSI, suggesting that the *BRAF-*mutated tumours are not uniform [42]. Further studies dissecting the immune response to *BRAF-*mutated tumours in MSI as well as MSS CRCs, including also analyses of immune checkpoint molecules, are needed to find potential predictive markers for immunotherapy. For patients with *BRAF-*mutated MSI CRCs, combined treatment with immune checkpoint inhibitors and targeted therapies are currently under clinical evaluation. In a clinical trial, Pembrolizumab as first line treatment of advanced MSI metastatic CRC showed good efficacy regardless of *BRAF* mutation [43]. However, a decreased benefit of Pembrolizumab treatment was found in *KRAS-* or *NRAS-*mutated metastatic MSI patients [43]. Furthermore, combination therapy with Nivolumab and Ipilimumab (CTLA4 inhibitor), showed evident beneficial effects, especially for patients with *BRAF-*mutated tumours [44]. Interestingly, even though limited by few patients, an improved clinical response to Pembrolizumab was found in patients with *BRAF-*mutated treatment-resistant metastatic MSS CRCs [45]. Importantly, our findings show that there are highly immune infiltrated MSS tumours (being the majority of CRCs), which should be further explored in terms of possible implications for immunotherapy. One limitation of this study was that immune analyses were restricted to patients who underwent primary surgery. The potential predictive value of immune markers needs to be further assessed also in the neo-adjuvant setting using larger patient cohorts. In fact, pathological responses have been shown for neo-adjuvant immunotherapy in both MSI and MSS early-stage cancers [46].

Mechanistic insights on the regulation of immunity according to *KRAS* and *BRAF* mutation are limited. In a study by Liao et al. *KRAS* mutation was found to direct the tumour recruitment of myeloid derived suppressor cells through the IRF2-CXCL3-CXCR2 axis leading to immune therapy resistance in CRC [39]. In a previous in vitro study comparing the cytokine profile of *KRAS* mutant, *BRAF* mutant, and *BRAF*/*KRAS* wild-type Caco2 CRC cells, we found an increased expression of a Th1 related cytokine (CXCL10), and decreased expression of Th2 related cytokines (CCL2 and TGFB1) in *BRAF-*mutated cells, while the opposite pattern was shown for *KRAS-*mutated cells [37]. The results were confirmed in tumour tissues and linked to infiltration of Th1 cells, suggesting that these mutations may have different and direct effects on the immune response to CRC.

In this study, we further analysed the prognostic role of immune cell infiltration in CRC tumours and found a significantly improved survival for patients with a high number of infiltrating T cell subsets (the strongest effect seen with cytotoxic T cells). The prognostic importance of cytotoxic T cells and regulatory T cells remained also in stage I-III patients.

*KRAS* mutation has been associated with a poor prognosis in CRC [47, 48], and so has *BRAF* mutation, irrespective of MSI status, with *BRAF-*mutated MSS tumours showing the worst prognosis [18]. Here, survival analyses of infiltrating cytotoxic T cells at the tumour front showed a strong prognostic role for cytotoxic T cells in CRC independent of *KRAS-* and *BRAF-*mutational status. Further studies are needed to understand the prognostic importance of immune cell infiltration in these molecular subgroups of CRC.

The strengths of this study include the quantitative evaluation of multiple immune cells in different tumour locations of patients with CRC, comprehensive immune evaluations on a protein-based level being rare and an important complement to the immunogenomic studies. We were also able to validate our main findings in an independent larger cohort of CRC patients. Weaknesses of this study include small sample sizes in the primary patient cohort and the possibility that the use of TMAs do not fully reflect the heterogenous distribution of tumour infiltrating immune cells within the whole tumour. Furthermore, the analyses in this study were restricted to *BRAF*^*V600E*^ mutation and *KRAS* mutations in codon 12 and 13. Even though covering the majority of *KRAS* and *BRAF* mutations in CRC, further studies taking additional mutations into account are needed.

Collectively, our findings suggest that combined evaluation of MSI status, *KRAS* and *BRAF* mutation, and immune cell infiltration (cytotoxic T cells) may provide added value compared to each variable alone and might have potential to help guide decisions on immunotherapy in CRC. In future management of CRC patients, individualised therapy taking both the molecular characteristics of the tumour and the tumour immune response into consideration will be of importance. An increased understanding of the actions of the immune response in molecular subgroups of CRC may lead to significant advances in personalised medicine, including the identification of important prognostic and predictive tools, as well as new targets for therapy.

### Supplementary information

Supplemental Information

REMARK checklist

## Data Availability

The data presented in this study are available on reasonable request from the corresponding author.

## References

[1] Morgan E, Arnold M, Gina A, Lorenzoni V, Cabasag ML, Laversanne M (2023). Global burden of colorectal cancer in 2020 and 2040: incidence and mortality estimates from GLOBOCAN. Gut..

[2] Galon J, Costes A, Sanchez-Cabo F, Kirilovsky A, Mlecnik B, Lagorce-Pages C (2006). Type, density, and location of immune cells within human colorectal tumors predict clinical outcome. Science..

[3] Galon J, Mlecnik B, Bindea G, Angell HK, Berger A, Lagorce C (2014). Towards the introduction of the ‘Immunoscore’ in the classification of malignant tumours. J Pathol.

[4] Deschoolmeester V, Baay M, Lardon F, Pauwels P, Peeters M (2011). Immune cells in colorectal cancer: prognostic relevance and role of MSI. Cancer Microenviron.

[5] Palucka AK, Coussens LM (2016). The basis of oncoimmunology. Cell.

[6] Le DT, Durham JN, Smith KN, Wang H, Bartlett BR, Aulakh LK (2017). Mismatch repair deficiency predicts response of solid tumors to PD-1 blockade. Science.

[7] Poh A (2022). A wow for Neoadjuvant ICI in dMMR colon cancer. Cancer Discov.

[8] Cercek A, Lumish M, Sinopoli J, Weiss J, Shia J, Lamendola-Essel M (2022). PD-1 blockade in mismatch repair-deficient, locally advanced rectal cancer. N. Engl J Med.

[9] Baldus SE, Schaefer KL, Engers R, Hartleb D, Stoecklein NH, Gabbert HE (2010). Prevalence and heterogeneity of KRAS, BRAF, and PIK3CA mutations in primary colorectal adenocarcinomas and their corresponding metastases. Clin Cancer Res..

[10] Randrian V, Evrard C, Tougeron D (2021). Microsatellite instability in colorectal cancers: carcinogenesis, neo-antigens, immuno-resistance and emerging therapies. Cancers.

[11] Mlecnik B, Bindea G, Angell HK, Maby P, Angelova M, Tougeron D (2016). Integrative analyses of colorectal cancer show immunoscore is a stronger predictor of patient survival than microsatellite instability. Immunity.

[12] Domingo E, Freeman-Mills L, Rayner E, Glaire M, Briggs S, Vermeulen L (2016). Somatic POLE proofreading domain mutation, immune response, and prognosis in colorectal cancer: a retrospective, pooled biomarker study. Lancet Gastroenterol Hepatol.

[13] Li X, Ling A, Kellgren TG, Lundholm M, Lofgren-Burstrom A, Zingmark C (2020). A detailed flow cytometric analysis of immune activity profiles in molecular subtypes of colorectal cancer. Cancers.

[14] Nebot-Bral L, Brandao D, Verlingue L, Rouleau E, Caron O, Despras E (2017). Hypermutated tumours in the era of immunotherapy: the paradigm of personalised medicine. Eur J Cancer.

[15] Ahtiainen M, Wirta EV, Kuopio T, Seppala T, Rantala J, Mecklin JP (2019). Combined prognostic value of CD274 (PD-L1)/PDCDI (PD-1) expression and immune cell infiltration in colorectal cancer as per mismatch repair status. Mod Pathol.

[16] Lee LH, Cavalcanti MS, Segal NH, Hechtman JF, Weiser MR, Smith JJ (2016). Patterns and prognostic relevance of PD-1 and PD-L1 expression in colorectal carcinoma. Mod Pathol.

[17] Rajagopalan H, Bardelli A, Lengauer C, Kinzler KW, Vogelstein B, Velculescu VE (2002). Tumorigenesis: RAF/RAS oncogenes and mismatch-repair status. Nature.

[18] Lochhead P, Kuchiba A, Imamura Y, Liao X, Yamauchi M, Nishihara R (2013). Microsatellite instability and BRAF mutation testing in colorectal cancer prognostication. J Natl Cancer Inst.

[19] Cen S, Liu K, Zheng Y, Shan J, Jing C, Gao J (2021). BRAF mutation as a potential therapeutic target for checkpoint inhibitors: a comprehensive analysis of immune microenvironment in BRAF mutated colon cancer. Front Cell Dev Biol.

[20] Glimelius B, Melin B, Enblad G, Alafuzoff I, Beskow A, Ahlstrom H (2018). U-CAN: a prospective longitudinal collection of biomaterials and clinical information from adult cancer patients in Sweden. Acta Oncol.

[21] Lowenmark T, Lofgren-Burstrom A, Zingmark C, Eklof V, Dahlberg M, Wai SN (2020). Parvimonas micra as a putative non-invasive faecal biomarker for colorectal cancer. Sci Rep..

[22] Lowenmark T, Lofgren-Burstrom A, Zingmark C, Ljuslinder I, Dahlberg M, Edin S (2022). Tumour colonisation of parvimonas micra Is associated with decreased survival in colorectal cancer patients. Cancers.

[23] Renman D, Gylling B, Vidman L, Boden S, Strigard K, Palmqvist R (2021). Density of CD3(+) and CD8(+) cells in the microenvironment of colorectal cancer according to prediagnostic physical activity. Cancer Epidemiol Biomark Prev.

[24] Edin S, Kaprio T, Hagstrom J, Larsson P, Mustonen H, Bockelman C (2019). The prognostic importance of CD20(+) B lymphocytes in colorectal cancer and the relation to other immune cell subsets. Sci Rep..

[25] Sullivan BM, Juedes A, Szabo SJ, von Herrath M, Glimcher LH (2003). Antigen-driven effector CD8 T cell function regulated by T-bet. Proc Natl Acad Sci USA.

[26] Mezheyeuski A, Micke P, Martin-Bernabe A, Backman M, Hrynchyk I, Hammarstrom K, et al. The immune landscape of colorectal cancer. Cancers. 2021;13:5545. 10.3390/cancers13215545 PMC8583221 34771707

[27] Inamura K (2018). Colorectal cancers: an update on their molecular pathology. Cancers.

[28] Manda K, Glasow A, Paape D, Hildebrandt G (2012). Effects of ionizing radiation on the immune system with special emphasis on the interaction of dendritic and T cells. Front Oncol.

[29] Carvalho HA, Villar RC (2018). Radiotherapy and immune response: the systemic effects of a local treatment. Clinics..

[30] Lim SH, Chua W, Cheng C, Descallar J, Ng W, Solomon M (2014). Effect of neoadjuvant chemoradiation on tumor-infiltrating/associated lymphocytes in locally advanced rectal cancers. Anticancer Res.

[31] Matsutani S, Shibutani M, Maeda K, Nagahara H, Fukuoka T, Nakao S (2018). Significance of tumor-infiltrating lymphocytes before and after neoadjuvant therapy for rectal cancer. Cancer Sci.

[32] Sakuyama N, Kojima M, Kawano S, Akimoto T, Saito N, Ito M (2016). Histological differences between preoperative chemoradiotherapy and chemotherapy for rectal cancer: a clinicopathological study. Pathol Int.

[33] Angelova M, Charoentong P, Hackl H, Fischer ML, Snajder R, Krogsdam AM (2015). Characterization of the immunophenotypes and antigenomes of colorectal cancers reveals distinct tumor escape mechanisms and novel targets for immunotherapy. Genome Biol.

[34] Giannakis M, Mu XJ, Shukla SA, Qian ZR, Cohen O, Nishihara R (2016). Genomic correlates of immune-cell infiltrates in colorectal carcinoma. Cell Rep..

[35] van den Bulk J, Verdegaal EME, Ruano D, Ijsselsteijn ME, Visser M, van der Breggen R (2019). Neoantigen-specific immunity in low mutation burden colorectal cancers of the consensus molecular subtype 4. Genome Med.

[36] Lal N, Beggs AD, Willcox BE, Middleton GW (2015). An immunogenomic stratification of colorectal cancer: implications for development of targeted immunotherapy. Oncoimmunology.

[37] Ling A, Lundberg IV, Eklof V, Wikberg ML, Oberg A, Edin S (2016). The infiltration, and prognostic importance, of Th1 lymphocytes vary in molecular subgroups of colorectal cancer. J Pathol Clin Res.

[38] Lal N, White BS, Goussous G, Pickles O, Mason MJ, Beggs AD (2018). KRAS mutation and consensus molecular Subtypes 2 and 3 are independently associated with reduced immune infiltration and reactivity in colorectal cancer. Clin Cancer Res.

[39] Liao W, Overman MJ, Boutin AT, Shang X, Zhao D, Dey P (2019). KRAS-IRF2 axis drives immune suppression and immune therapy resistance in colorectal cancer. Cancer Cell.

[40] Liu J, Huang X, Liu H, Wei C, Ru H, Qin H (2021). Immune landscape and prognostic immune-related genes in KRAS-mutant colorectal cancer patients. J Transl Med.

[41] Bolzacchini E, Libera L, Church SE, Sahnane N, Bombelli R, Digiacomo N (2022). Tumor antigenicity and a pre-existing adaptive immune response in advanced BRAF mutant colorectal cancers. Cancers.

[42] Barras D, Missiaglia E, Wirapati P, Sieber OM, Jorissen RN, Love C (2017). BRAF V600E mutant colorectal cancer subtypes based on gene expression. Clin Cancer Res.

[43] Andre T, Shiu KK, Kim TW, Jensen BV, Jensen LH, Punt C (2020). Pembrolizumab in microsatellite-instability-high advanced colorectal cancer. N. Engl J Med.

[44] Andre T, Lonardi S, Wong KYM, Lenz HJ, Gelsomino F, Aglietta M (2022). Nivolumab plus low-dose ipilimumab in previously treated patients with microsatellite instability-high/mismatch repair-deficient metastatic colorectal cancer: 4-year follow-up from CheckMate 142. Ann Oncol.

[45] Wang C, Sandhu J, Ouyang C, Ye J, Lee PP, Fakih M (2021). Clinical response to immunotherapy targeting programmed cell death receptor 1/programmed cell death Ligand 1 in patients with treatment-resistant microsatellite stable colorectal cancer with and without liver metastases. JAMA Netw Open.

[46] Chalabi M, Fanchi LF, Dijkstra KK, Van den Berg JG, Aalbers AG, Sikorska K (2020). Neoadjuvant immunotherapy leads to pathological responses in MMR-proficient and MMR-deficient early-stage colon cancers. Nat Med.

[47] Imamura Y, Morikawa T, Liao X, Lochhead P, Kuchiba A, Yamauchi M (2012). Specific mutations in KRAS codons 12 and 13, and patient prognosis in 1075 BRAF wild-type colorectal cancers. Clin Cancer Res.

[48] Modest DP, Ricard I, Heinemann V, Hegewisch-Becker S, Schmiegel W, Porschen R (2016). Outcome according to KRAS-, NRAS- and BRAF-mutation as well as KRAS mutation variants: pooled analysis of five randomized trials in metastatic colorectal cancer by the AIO colorectal cancer study group. Ann Oncol.

