# Supplementary material for: Opposing roles by KRAS and BRAF mutation on immune cell infiltration in colorectal cancer – possible implications for immunotherapy
Source: Br J Cancer. 2023 Dec 1;130(1):143–50. doi: 10.1038/s41416-023-02483-9 (PMC10781968; doi:10.1038/s41416-023-02483-9)
Supplement: Supplementary file 1 — Supplemental Information [file 41416_2023_2483_MOESM1_ESM.pdf]

SUPPLEMENTARY INFORMATION

Supplementary Table S1: Clinical characteristics of CRC patients from NSHDS

Supplementary Table S2: Concentrations of antibodies and Opal dyes

Supplementary Table S3: Associations of immune cells in tumour centre to clinicopathological characteristics of CRC patients

Supplementary Table S4: Associations of immune cells in tumour centre to molecular characteristics of CRC tumours

Supplementary Table S5: Multivariable Cox regression analyses in stage I-III patients from the NSHDS cohort

Supplementary Figure S1: The prognostic importance of infiltrating immune cells at the tumour front in CRC

Supplementary Table S1. Clinical characteristics of CRC patients from NSHDS

Frequency, n (%)	608 (100.0)
Gender, n (%)	
Man	272 (44.7)
Woman	336 (55.3)
Age, n (%)	
≤ 59	110 (18.1)
60-69	235 (38.7)
70-79	228 (37.5)
≥ 80	35 (5.8)
Localisation, n (%)	
Right colon	242 (39.8)
Left colon	192 (31.6)
Rectum	174 (28.6)
Stage, n (%)	
I	137 (22.5)
II	196 (32.2)
III	168 (27.6)
IV	106 (17.4)
Pre-operative RT, n (%)	
No	510 (83.9)
Yes	98 (16.1)

Abbreviations: RT, radiotherapy

Supplementary Table S2. Concentration of antibodies and Opal dyes.

Antigen	Final concentration (µg/ml)	Opal Dye	Opal dilution
T-bet	4	520	1/100
CD20	5	540	1/200
CD8	0.1	570	1/100
FoxP3	0.3	620	1/100
CD68	0.2	650	1/150
Cytokeratin	3.6	690	1/50

Supplementary Table S3. Associations of immune cells in tumour centre to clinicopathological characteristics of CRC patients.

Tumour centre										
Stromal area								Tumour epithelial area		
		n	Cytotoxic T cells	T helper 1 cells	Regulatory T cells	B cells	Macrophages	Cytotoxic T cells	T helper 1 cells	Macrophages
Gender	Man	67								
	Woman	70								
	P value		0.381	0.109	0.187	0.009*	0.181	0.340	0.170	0.034*
Age	<=59	24								
	60-69	52								
	70-79	41								
	>=80	20								
	P value		0.039*	0.035*	0.313	0.236	<0.001*	0.042*	0.016*	0.292
Localisation	Right colon	38								
	Left colon	26								
	Rectum	73								
	P value		<0.001*	0.099	0.226	0.386	0.087	<0.001*	0.021*	0.244
Stage	I	28								
	II	52								
	III	44								
	IV	12								
	P value		0.040*	0.073	0.153	0.044*	0.049*	0.009*	0.295	0.336
Grade	Low grade	115								
	High grade	19								
	P value		0.030*	0.499	0.194	0.515	0.173	0.134	0.437	0.523
Mucinous	Non-mucinous	118								
	Mucinous	16								
	P value		0.232	0.805	0.184	0.941	0.271	0.346	0.677	0.638
Pre-operative RT	No	92								
	Yes	45								
	P value		<0.001*	0.133	0.187	0.950	0.110	<0.001*	0.258	0.135
			0-600					0-60		

Shown are median numbers of infiltrating cells/mm^2 stromal tissue or tumour epithelial tissue within the tumour centre. Abbreviation: RT, radiotherapy.
*Indicates significant *P* values (P < 0.05) according to Mann-Whitney *U* or Kruskall-Wallis *H* tests.

Supplementary Table S4. Associations of immune cells in tumour centre to molecular characteristics of CRC tumours.

Tumour centre										
Stromal area								Tumour epithelial area		
			Cytotoxic T cells	T helper 1 cells	Regulatory T cells	B cells	Macrophages	Cytotoxic T cells	T helper 1 cells	Macrophages
KRAS status	wild-type	60								
	mutant	29								
	P value		0.276	0.398	0.017*	0.224	0.979	0.040*	0.369	0.274
BRAF status	wild-type	74								
	mutant	18								
	P value		0.555	0.095	0.622	0.633	0.081	0.199	0.053	0.031*
KRAS/BRAF status	wild-type/wild-type	43								
	KRAS mutant	29								
	BRAF mutant	17								
	P value		0.500	0.136	0.051	0.437	0.299	0.084	0.085	0.049*
MSI status	MSS	76								
	MSI	16								
	P value		0.157	0.011*	0.156	0.584	0.042*	0.059	0.003*	0.045*
MSI status/*BRAF* status	MSS *BRAF* wild-type	69								
	MSS *BRAF* mutant	7								
	MSI *BRAF* wild-type	5								
	MSI *BRAF* mutant	11								
	P value		0.554	0.088	0.539	0.735	0.081	0.253	0.021*	0.067
			0-600					0-120		

Pre-operatively irradiated rectal cancers were excluded. Shown are median numbers of infiltrating cells/mm^2 stromal tissue or tumour epithelial tissue within the tumour centre. *Indicates significant *P* values ($P < 0.05$) according to Mann-Whitney *U* tests or Kruskall-Wallis *H* tests.

Supplementary Table S5. Multivariable Cox regression analyses in stage I-III patients from the NSHDS cohort.

		Univariable			Multivariable		
		HR	95% CI	*P* value	HR	95%CI	*P* value
Gender	Kvinna	1	-	-	1	-	-
	Man	0.76	0.47 -1.23	0.259	0.75	0.41-1.39	0.364
Age	<=59	1	-	-	1	-	-
	60-69	0.92	0.47-1.80	0.802	1.15	0.50-2.66	0.741
	70-79	1.10	0.57-2.12	0.789	1.58	0.67-3.74	0.296
	>=80	1.95	0.78-4.92	0.155	1.55	0.53-4.59	0.427
Localisation	Right colon	1	-	-	1	-	-
	Left colon	1.28	0.77-2.14	0.341	1.53	0.73-3.19	0.256
	Rectum	1.41	0.74-2.68	0.296	3.04	1.28-7.25	0.012
Stage	I	1	-	-	1	-	-
	II	2.26	0.96-5.32	0.062	2.58	0.82-8.14	0.106
	III	6.62	2.98-14.67	<0.001	9.01	3.10-26.22	<0.001
KRAS status	Wild-type	1	-	-	1	-	-
	Mutant	1.10	0.65-1.87	0.717	1.29	0.67-2.46	0.445
BRAF status	Wild-type	1	-	-	1	-	-
	mutant	1.57	0.97-2.55	0.068	2.68	1.21-5.95	0.015
MSI status	MSS	1	-	-	1	-	-
	MSI	0.84	0.46-1.54	0.570	0.84	0.31-2.26	0.723
Front stroma CD8	1	1	-	-	1	-	-
	2	0.39	0.22-0.70	0.002	0.29	0.15-0.58	<0.001
	3	0.33	0.17-0.64	0.001	0.24	0.11-0.55	<0.001
	4	0.28	0.12-0.65	0.003	0.32	0.12-0.84	0.021

Abbreviations: HZ, hazard ratio; CI, confidence interval.

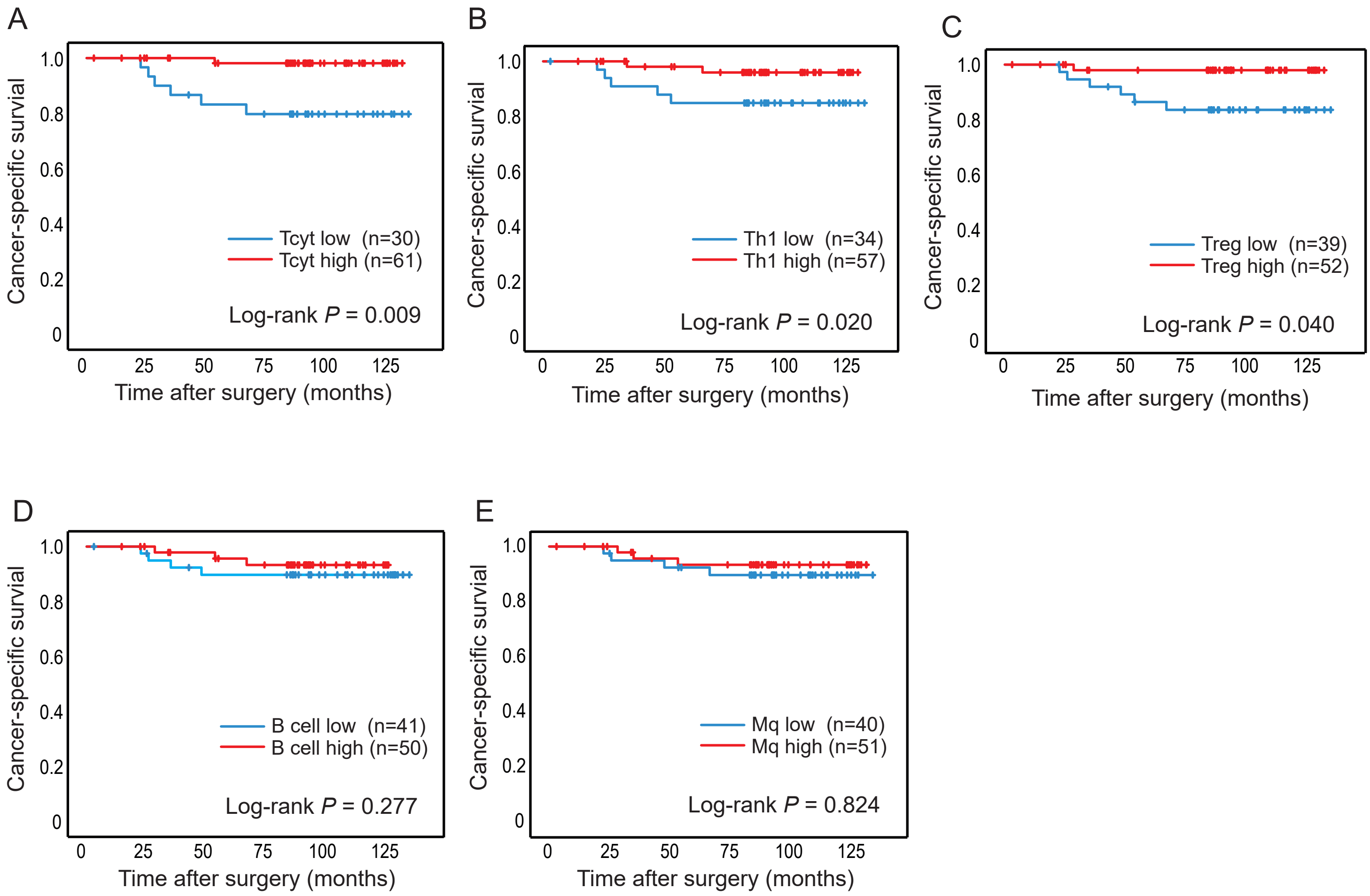

Supplementary Figure S1. The prognostic importance of infiltrating immune cells at the tumour front in CRC. Shown are Kaplan-Meier plots of cancer-specific survival in patients with low or high stromal infiltration of (A) cytotoxic T cells (Tcyt), (B) T helper 1 cells (Th1), (C) T regulatory cells (Tregs), (D) B cells, and (E) macrophages (Mq). Log-rank tests were used to calculate differences in 5-year survival between groups. $P < 0.05$ was considered statistically significant.
